# Prevalence of pectus excavatum (PE), pectus carinatum (PC), tracheal hypoplasia, thoracic spine deformities and lateral heart displacement in thoracic radiographs of screw-tailed brachycephalic dogs

**DOI:** 10.1371/journal.pone.0223642

**Published:** 2019-10-10

**Authors:** Renata Komsta, Zbigniew Osiński, Piotr Dębiak, Piotr Twardowski, Barbara Lisiak

**Affiliations:** 1 Laboratory for Radiology and Ultrasonography Department and Clinic of Animal Surgery, Faculty of Veterinary Medicine, University of Life Science, Lublin, Poland; 2 National Veterinary Research Institute, Puławy, Poland; University of Bari, ITALY

## Abstract

Pectus excavatum, thoracic spine deformities, tracheal hypoplasia and lateral heart displacement are frequently described in brachycephalic dog breeds. Pectus carinatum is described sporadically, although the authors' observations demonstrate that it may occur in certain brachycephalic dog breeds. It was hypothesised that dogs of screw-tailed brachycephalic breeds carry a greater risk of these anomalies than normal-tailed brachycephalic breeds, and that there could a relation between the presence of pectus excavatum or pectus carinatum and thoracic spine deformities, tracheal hypoplasia and lateral heart displacement. During retrospective studies, these anomalies were identified in lateral and dorso-ventral radiographs of the thorax in brachycephalic dog breeds. A statistical analysis revealed that the frequency of pectus excavatum occurrence in screw-tailed and normal-tailed brachycephalic dog breeds is similar. The greatest risk of pectus excavatum occurrence is carried by two breeds: Maltese (60%) and English Bulldog (58%), while for pectus carinatum: Pug (41%) and French Bulldog (18%). Dogs of screw-tailed brachycephalic breeds carry a greater risk of kyphosis (p < 0.0001), tracheal hypoplasia occurrence (p < 0.0001), compared to "normal-tailed" breeds. The hypothesis concerning a relation between the presence of pectus excavatum or pectus carinatum and the other anomalies studied was not confirmed (p > 0.05). It was demonstrated that in dogs of brachycephalic breeds there was a greater risk of co-incidence between kyphosis of the thoracic spine and lateral heart displacement (p = 0.038), as well as kyphosis of the thoracic spine and tracheal hypoplasia (p = 0.003).

## Introduction

Pectus excavatum (PE) is an anomaly of the chest wall, characterized by the dorsal deviation of the sternum and associated costal cartilages with subsequent a dorsoventral flattening of the entire thorax [1–7]. PE is classified as a congenital disorder, although in 2012 Kurosawa reported an acquired form of PE in a 13 year old Labrador-Retriever [8]. This disorder is not frequently observed in animals, although it is particularly frequently reported in brachycephalic dog breeds. Pectus carinatum (PC) is characterized by protrusion of the sternum [2, 9]. It is a sporadically reported congenital disorder of the thoracic wall. Both defects may remain symptomless, although PE may lead to severe dyspnoea and abnormalities of the cardiovascular function, while PC may cause transient respiratory distress. Diagnosing PE and PC is based on clinical and radiological examinations [7, 9].

Pectus excavatum and pectus carinatum are also reported in humans, and are believed to be connected with the occurrence of vertebral deformities, in particular with scoliosis [10–12]. Kyphosis and scoliosis also occurs in dogs, in particular in screw-tailed brachycephalic breeds [13, 14]. In animals, PE is frequently found alongside tracheal hypoplasia [1, 4], cardiomegaly, and malposition of the heart [1, 5, 7, 15].

The purpose of this retrospective study was to evaluate the prevalence of PE and PC, thoracic spine deformities, tracheal hypoplasia and lateral heart displacement in lateral and dorso-ventral thoracic radiographs in screw-tailed and normal-tailed brachycephalic dog breeds. The authors hypothesised that dogs of screw-tailed brachycephalic breeds carried a greater risk of the occurrence of the above anomalies than normal-tailed brachycephalic dog breeds. Our secondary hypothesis was that there was a statistically significant relation between the presence of PE or PC, and thoracic spine deformities, tracheal hypoplasia and lateral heart displacement.

## Material and methods

### Ethics statement

In accordance with the Act “On the Protection of Animals Used for Scientific or Educational Purposes” (15/01/2015) this study was considered as sub-threshold for specific ethical approval, as the work involved only the analysis of data routinely recorded from normal and necessary clinical procedures.

### Cases

The digital medical database of the Faculty of Veterinary Medicine at University of Life Sciences in Lublin was retrospectively reviewed from March 2011 and September 2018. The criteria for inclusion in the study were as follows: dogs of selected brachycephalic breeds (English Bulldog (EB), French Bulldog (FB), Pug (Pu), Maltese (Mlt), Cavalier King Charles Spaniel (Cav), Shih Tzu (Sht) and Pekingese (Pkn)) with lateral and dorso-ventral radiographs covering the entire thorax. The criteria for exclusion were as follows: dogs diagnosed with diaphragmatic hernia, a very large volume of unilateral pleural gas/fluid and a pericardial tamponade. For each dog, which met the inclusion criteria, the breed, age, and sex were recorded. These dogs were then assigned to one of two groups, based on their breed: Group A: screw-tailed brachycephalic breeds (n = 68) including EB, FB and Pu [14], and Group B: normal-tailed brachycephalic breeds (n = 56) including Mlt, Cav, Sht, and Pkn.

### Radiographic assessment

Thoracic radiographs were carried out using a digital radiography system (INTUITION HFe 6001, ARCOMA), and analysed by three board certified veterinary radiologists (RK, PD, PT) using a DICOM PACS DXR X-ray Acquisition Software workstation.

**Fig 1:**
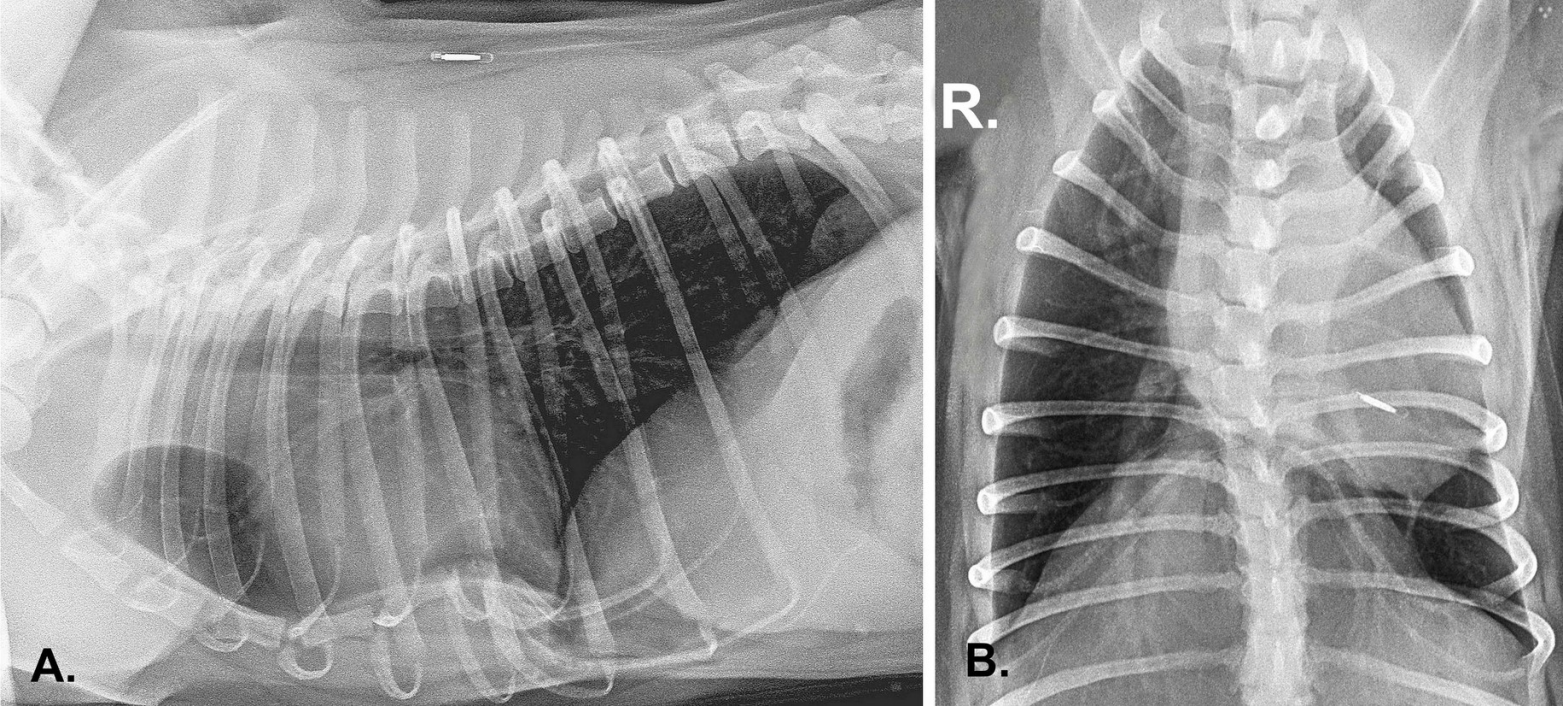
Right lateral (A) and dorso-ventral (B) thoracic radiograph of French bulldog with pectus excavatum, scoliosis and, left-sided heart displacement. There is the subluxation of the vertebrae (Th12/Th13).

**Fig 2:**
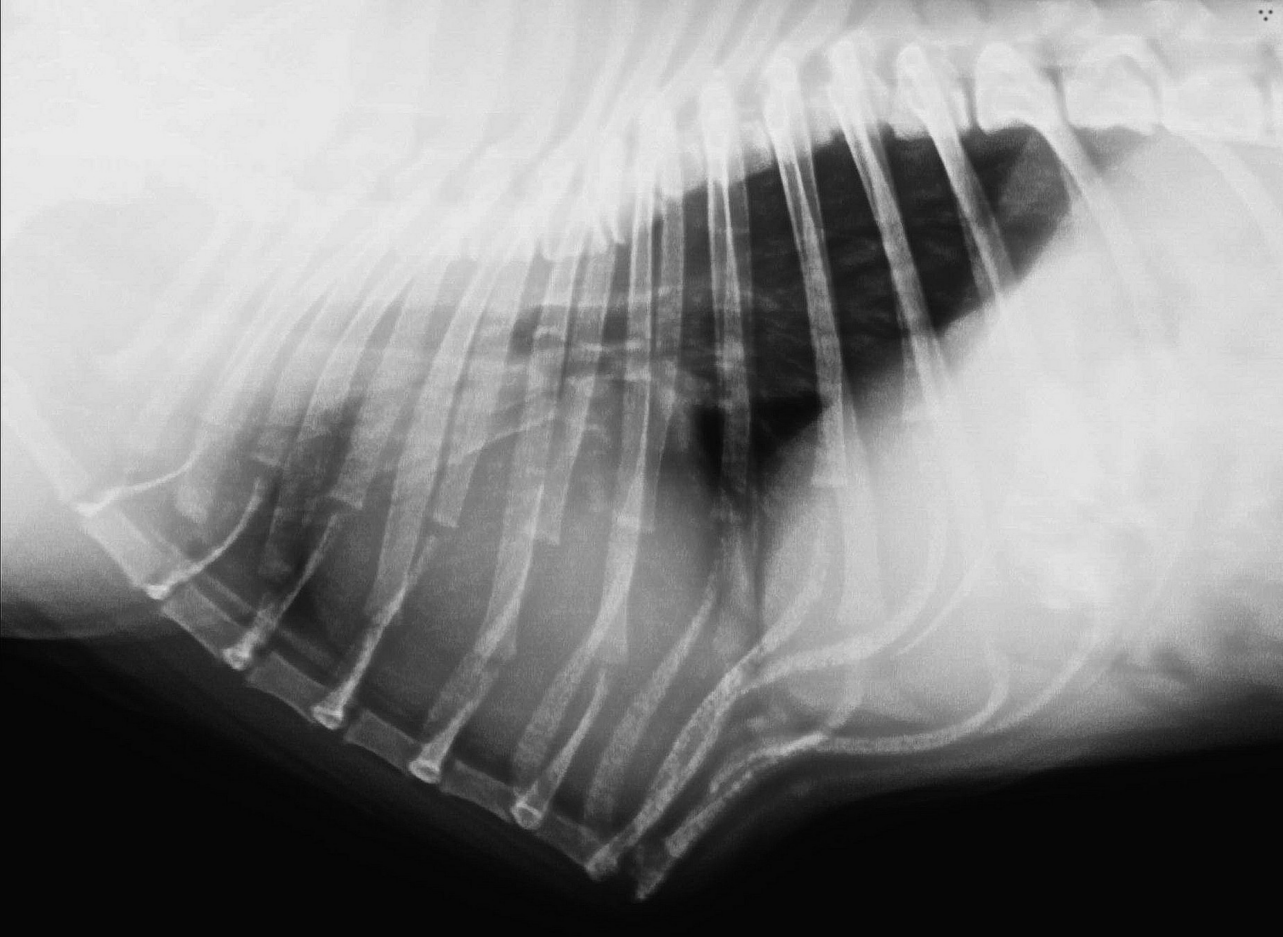
Right lateral thoracic radiograph of pug with pectus carinatum and kyphosis.

In each dog, the presence of the following qualitative characteristics was analysed: PE (broken down into types based on anatomic location of the defect, typical: ventrodorsal deviation of the sternum including the caudal sternum between 5^th^-8^th^ sternebrae /atypical: ventrodorsal deviation of the sternum encompassed the cranial or mid-sternal region) (Fig 1A) [3, 16], PC (Fig 2), kyphosis and scoliosis of the thoracic spine, tracheal hypoplasia, as well as right-sided and left-sided heart displacement.

**Fig 3:**
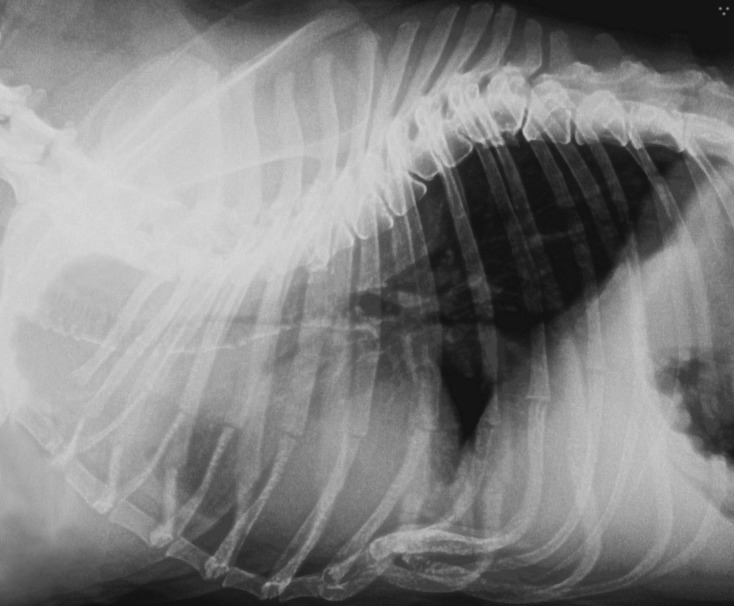
Right lateral thoracic radiograph of English bulldog with pectus excavatum and kyphosis.

**Fig 4:**
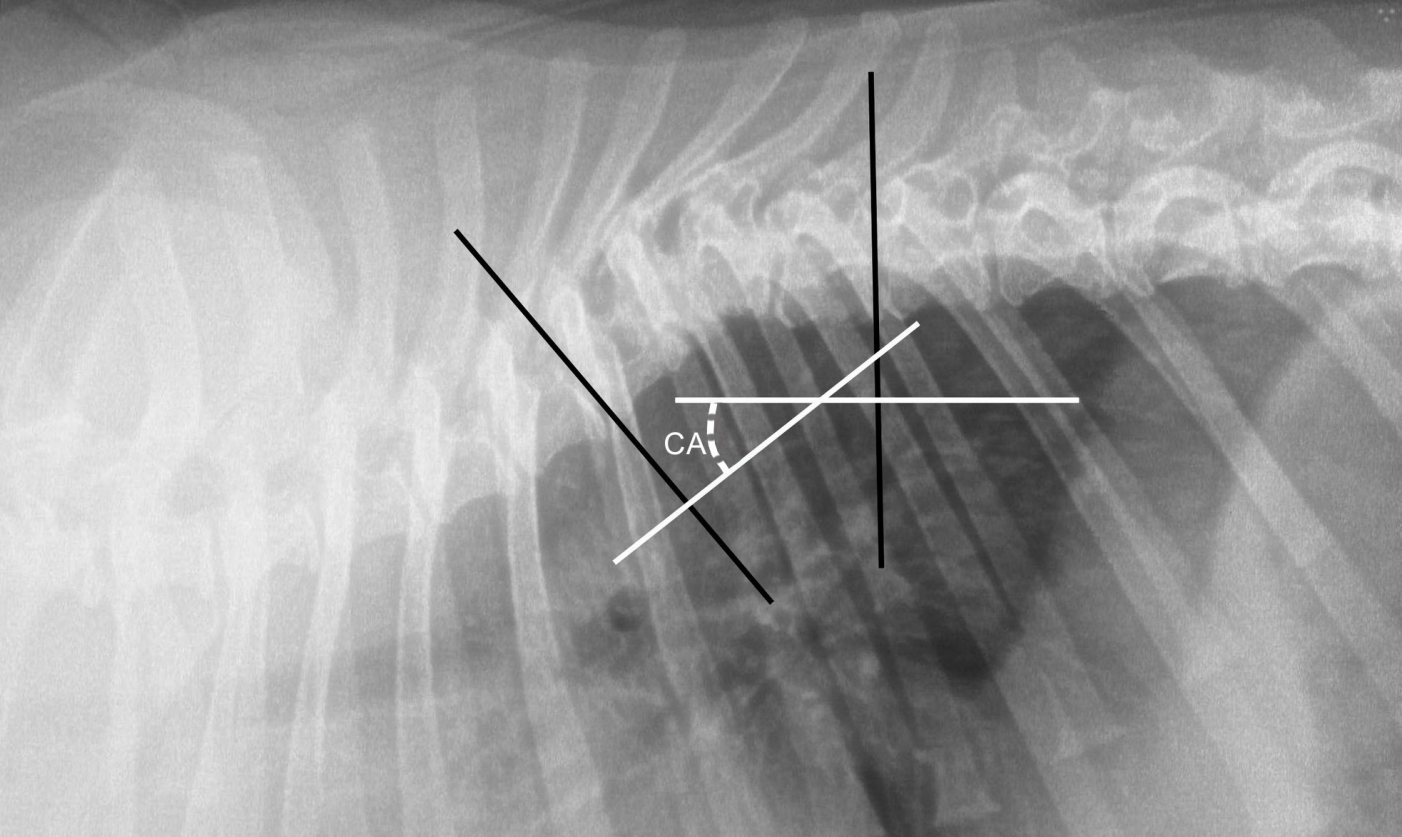
Lateral projection of thoracic vertebral column of a dog demonstrating measurement of the Cobb angle. CA–Cobb angle. To determine the Cobb angle, a line perpendicular to the cranial vertebral end plate of the first vertebra located cranialward of the malformed vertebra was drawn in the software. Similarly, a line was drawn perpendicular to the caudal vertebral end plate of the first vertebra located caudally to the malformed vertebra. The Cobb angle was measured at the point where the perpendicular lines crossed.

Kyphosis (Fig 3) was defined as dorsal spinal curvature with a Cobb angle exceeding 10 degrees on lateral radiographs [17, 18] (Fig 4). Scoliosis (Fig 1B) was defined as lateral vertebral angulation with a Cobb angle exceeding 10 degrees on the dorso-ventral radiograph of the thorax [17].

The trachea was analysed using lateral images of the thorax. The tracheal lumen diameter to thoracic inlet distance ratio was assessed. The presence of hypoplasia was indicated by a value lower than 0.09 for English Bulldogs and lower than 0.13 for dogs of the other studied breeds [19, 20].

Based on dorso-ventral images, the position of the heart was analysed. If cardiac silhouette and apex were located closer to the hemithorax and the cardiac outline was in contact with thoracic wall or close to the thoracic wall, the heart was considered to be displaced to the right or left side of the hemithorax (Fig 1B).

### Statistical analysis

To assess the relation between the selected thoracic anomalies (PE, PC, deformity of thoracic vertebrae, tracheal hypoplasia, heart displacement) in the study dogs (group A and B, breeds of dog) and factors which are conducive to their occurrence (deformity of thoracic vertebrae, tracheal hypoplasia, heart displacement), the Odds Ratio (OR) indicator was used. The OR statistics were calculated using either of two approaches, depending on the data available: the classic or the Bayesian. In the former, if the number of defect cases in each study dog group was sufficient to use the classic approach [21–24], the OR statistics were estimated and a chi squared test was performed for the difference between the position observed and the statistics calculated using the Mantel-Haenschel method as a correlated measure of risk. The *p-value* as the significance threshold was also calculated (*p*-value < 0.05 considered statistically significant). The calculations were made using R Foundation (ver 3.4) [25] statistical software and the "epir" module". The point estimators and confidence range estimation methods utilised in the module employed were based on equations described in the papers by Rothman [21] and Jewell [22]. In the latter approach to OR estimation, the Bayesian approach was used [26], where *a priori* data approximation using *Beta* distributions was applied.

Odds Ratio (OR) equation:

$$OR=\frac{P(Y=1|X=1)P(Y=0|X=0)}{P(Y=0|X=1)P(Y=1|X=0)}=\frac{\pi_{1}(1-\pi_{0})}{\pi_{0}(1-\pi_{1})}$$

where:

*π*_0_—probability of *posteriori* occurrence of defects in the group unaffected by the given risk factor, described by a *beta* distribution:

$$\pi_{0}⎸y∼beta(y_{0}+a_{0},n_{0}+b_{0})$$

*π*_1_—probability of *posteriori* occurrence of defects in the group affected by the given risk factor, described by a *beta* distribution:

$$\pi_{1}⎸y∼beta(y_{1}+a_{1},n_{1}+b_{1})$$

*A priori* inputs:

y_0_—number of dysfunctions found in the unaffected group,

n_0_—total number of dogs examined in the group unaffected by the risk factor (including both dysfunctional and defect-free cases),

y_1_—number of dysfunction cases for the group affected by the risk factor,

n_1_—total number of dogs studied in the group affected by the risk factor (including both dysfunctional and defect-free cases),

a_0_, a_1_, b_0_, b_1_—initial parameters in the Bayesian simulation using MCMC.

Due to a lack of previous information on the initial distribution of the input data, a_0_ = a_1 =_ b_0_ = b_1_ = 1, were assumed *a priori*, which means that the *beta* distributions took on a special form of a uniform distribution before the simulation. To analyse the impact of the dog breed on the occurrence of thoracic defects, correspondence analysis (CA) was used, a multidimensional exploration method [27–29]. The between-group differences were assessed by using χ2 (chi squared) statistical tests for categorical variables. To determine the statistical significance and strength of the relations between the analysed variables, F statistic (Fisher) values were analysed, accepting values of F > 1.00 at significance level at least p ≤ 0.05 for such comparisons. The correspondence analysis results are presented in a graphical form. The survey data were statistically analysed using the correspondence analysis method and Statistica 10.0 software.

## Results

124 dogs met the required criteria and were qualified for testing. A total of 68 dogs (34 females and 34 males) aged from 3 weeks to 15 years (mean age 4.2 years) were classified as screw-tailed dogs (group A). A total of 56 dogs (30 females and 26 males) aged from 3 weeks to 15 years (mean age 5.7 years) were classified as normal-tailed dogs (group B).

Imaging findings on dorso-ventral and lateral thorax radiographs from groups A and B are shown in Table 1. The prevalence of morphological traits in screw-tailed and normal-tailed dogs is shown in Table 2. Sporadic occurrences of selected anomalies in the studied dog population ruled them out from statistical assessment of mutual occurrence relation.

**Table 1:** Imaging findings on radiographs of thorax in the "screw-tailed" dog (A) group (*n* = 68) and the "normal-tailed" dog (B) group (n = 56).

Disorders		PE	PC	Kyphosis	Kypho-scoliosis	Scoliosis	Tracheal hypoplasia	Left-sided heart displacement	Right-sided heart displacement
A group (68)	EB (12)	7 (58.3%)	0 (0.0%)	7 (58.3%)	0 (0.0%)	1 (8.3%)	4 (33.3%)	3 (25.0%)	0 (0.0%)
	FB (39)	16 (41.0%)	7 (18.0%)	19 (48.7%)	2 (5.1%)	1 (2.6%)	15 (38.5%)	2 (5.1%)	1 (2.6%)
	Pu (17)	5 (29.4%)	7 (41.2%)	3 (17.6%)	0 (0.0%)	0 (0.0%)	8 (50.0%)	0 (0.0%)	0 (0.0%)
B group (56)	Mlt (10)	6 (60.0%)	0 (0.0%)	0 (0.0%)	0 (0.0%)	0 (0.0%)	2 (20.0%)	0 (0.0%)	0 (0.0%)
	Cav (10)	4 (40.0%)	0 (0.0%)	0 (0.0%)	0 (0.0%)	0 (0.0%)	1 (10.0%)	0 (0.0%)	0 (0.0%)
	Sht (17)	8 (47.1%)	0 (0.0%)	0 (0.0%)	0 (0.0%)	0 (0.0%)	1 (5.9%)	2 (11.8%)	0 (0.0%)
	Pkn (19)	9 (47.4%)	0 (0.0%)	1 (5.3%)	0 (0.0%)	0 (0.0%)	0 (0.0%)	1 (5.3%)	0 (0.0%)

PE, pectus excavatum; PC, pectus carinatum; EB, English Bulldog; FB, French Bulldog; Pu, Pug; Mlt, Maltese; Cav, Cavalier King Charles Spaniel; Sht, Shih Tzu; Pkn, Pekingese.

**Table 2:** Prevalence of morphological traits reported as number of dogs (%) for the "screw-tailed" dog (A) group (n = 68) and the "normal-tailed" dog (B) group (n = 56).

Variable	A group (%)	B group (%)	OR	p-value
PE	28 (41.2)	27 (48.2)	1.08 (0.74–1.56)	0.704
PC	14 (20.6)	0	88.99 (1.82–316.92)	B
Kyphosis	29 (42.6)	1 (1.8)	25.38 (3.57–180.32)	<0.0001*)
Kyphoscoliosis	2 (2.9)	0	47.61 (0.57–170.33)	B
Scoliosis	2 (2.9)	0	47.61 (0.57–170.33)	B
Tracheal hypoplasia	27 (39.7)	4 (7,1)	5.64 (2.10–15.16)	<0.0001*)
Left-sided heart displacement	5 (7.4)	3 (5,4)	1.39 (0.34–5.57)	0.639
Right-sided heart displacement	1 (1.5)	0	40.87 (0.15–65.47)	B

B - Bayesian approach. The posterior median and 95% credibility intervals.

*) level of significance at p < 0.05

PE was detected in 55 (44.4%) of the study animals: 28 in group A dogs (41.2%) and 27 in group B dogs (48.2%). In all cases, PE was observed between the 5th and 8th sternebra, which are typical locations. PE most commonly occurred in Maltese and English Bulldogs.

PC was observed in 14 (11.3%) of the study dogs, in 41.2% of the Pugs, 18% of the French Bulldogs and in none of the other breeds. The Bayesian statistical analysis indicated a possible greater risk of PC occurrence in screw-tailed breeds. However, the low number of study animals made it impossible to determine the multiplicity of PC occurrence risk.

**Fig 5:**
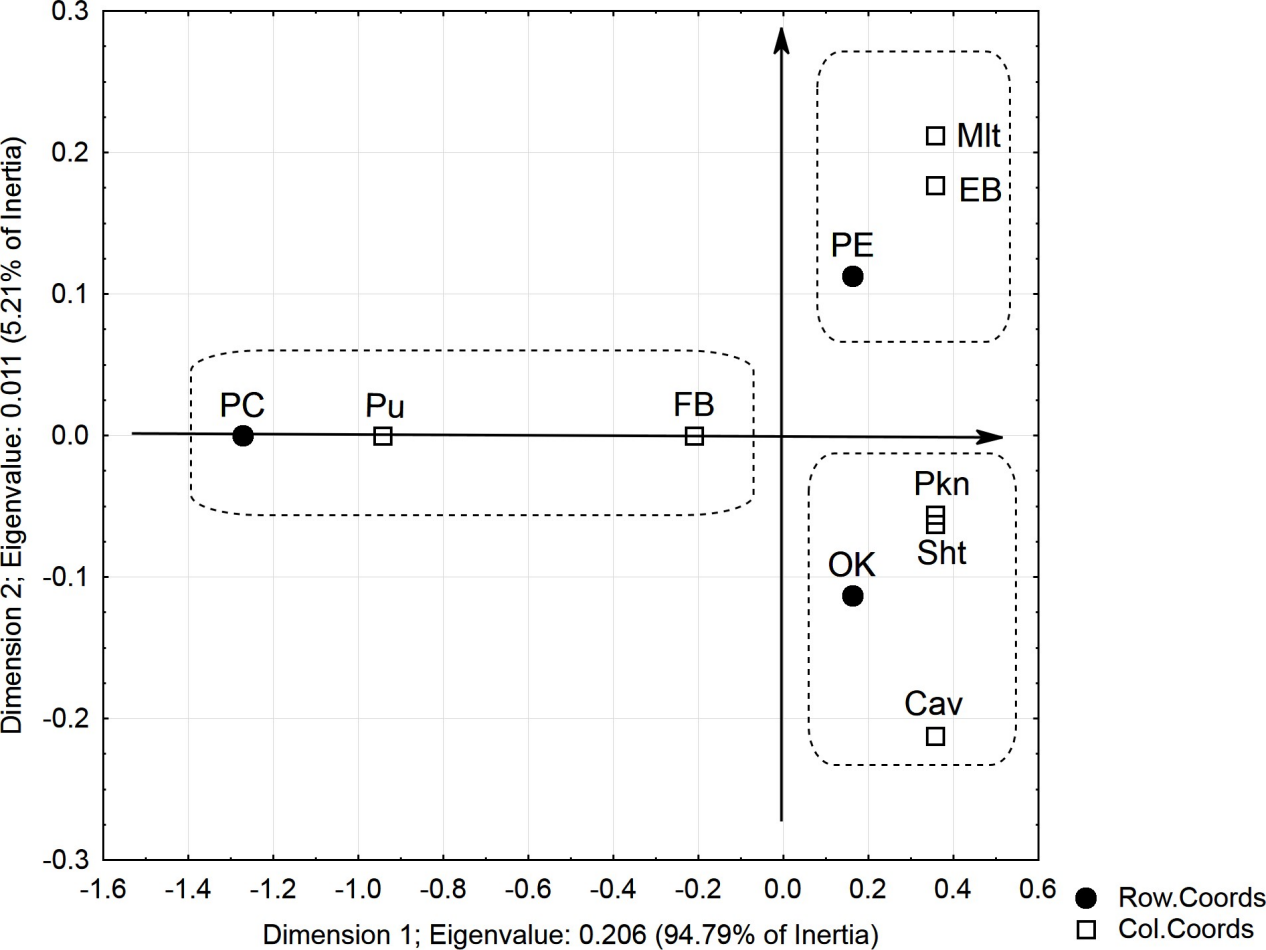
Graphical presentation of the relationship between dog breed and PE and PC.

The statistical analysis demonstrated a strong and statistically significant relation between the breed of dog and the risk of PE or PC occurrence (Chi-square = 26.94, p-Value = 0.0079) (Fig 5). The breeds at particular risk of PE occurrence were the Maltese and the English Bulldog, and at risk of PC were the Pug and the French Bulldog.

Thoracic spine deformities were found in 34 (27.4%) of brachycephalic dogs. The most common thoracic spine deformity was kyphosis, which accounted for 88.2% of all deformities found. Kyphosis was more commonly diagnosed in screw-tailed brachycephalic dogs. Its risk occurrence in this dog group was more than 25 times greater than in the normal-tailed brachycephalic dog group (Table 2). The defect was diagnosed in 58.3% of English Bulldogs and 48.7% of French Bulldogs. A correct shape of thoracic spine section was found in the Maltese, King Charles Spaniel and Shih Tzu (Table 1).

Tracheal hypoplasia was diagnosed in 31 dogs (25% of the study population), more commonly in screw-tailed breeds, especially in Pugs (50% of the breed). A strong and statistically significant relation between breed type and tracheal hypoplasia occurrence was demonstrated. Screw-tailed brachycephalic dogs were over 5 times more likely to suffer from this deformity than normal-tailed brachycephalic dogs (Table 2).

In 9 (7.3%) of the study dogs, lateral heart displacement was found (Table 1), in most cases (88.9%) it was displaced to the left side of the chest.

Statistical analysis failed to confirm a relation between PE or PC presence in dogs of brachycephalic breeds and the presence of thoracic spine kyphosis, tracheal hypoplasia or left-sided cardiac malposition (Table 3). No statistically significant relation was found between tracheal hypoplasia and left-sided cardiac malposition. However, a statistically significant relation between thoracic spine kyphosis and tracheal hypoplasia, as well as left-sided heart displacement was demonstrated.

**Table 3:** Odds Ratio (OR) for selected disorders associated with kyphosis, tracheal hypoplasia or left-sided heart displacement.

Disorders	Variable		Exposure		OR (CI)	p-value
			+	-		
PE	kyphosis	with	12	12	1.02 (0.50–2.06)	0.959
		without	41	42		
	tracheal hypoplasia	with	11	14	0.85 (0.52–1.38)	0.497
		without	44	41		
	left-sided heart displacement	with	6	2	1.58 (1.01–2.47)	0.136
		without	48	53		
PC	kyphosis	with	6	12	2.08 (0.96–4.49)	0.083
		without	7	42		
	tracheal hypoplasia	with	6	14	2.06 (0.79–5.36)	0.144
		without	7	41		
	left-sided heart displacement	with	0	2	2.42 (0.03–12.22)	B
		without	14	53		
Kyphosis	tracheal hypoplasia	with	14	17	2.48 (1.38–4.48)	0.003*)
		without	16	72		
	left-sided heart displacement	with	4	3	2.56 (1.24–5.31)	0.038*)
		without	25	87		
Tracheal hypoplasia	left-sided heart displacement	with	3	5	1.76 (0.45–6.95)	0.418
		without	28	86		

B - Bayesian approach. The posterior median and 95% credibility intervals.

*) level of significance at p < 0,.05

## Discussion

As far as the authors are aware, this paper is the first published attempt to evaluate the prevalence of PE and PC visible in lateral and dorso-ventral thoracic radiographs in brachycephalic breeds of dog. The issue of congenital thoracic wall defects in animals is relatively rarely addressed in veterinary literature. The most commonly described cases are those relate to moderate to severe respiratory distress [6–9, 15, 30, 31]. Charlesworth (2012) published a paper concerning the frequency of PE occurrence in Bengal cats. Based on clinical examinations of several week old cats of this breed, he assessed the prevalence of PE at 2% [32]. The authors of this paper have found no such papers concerning dogs. The results demonstrated the occurrence of PE in 44.4% of the dogs examined. Such a high percentage of animals with PE diagnosed likely stems from several factors, the breed type being particularly important. In 1989, Fossum (1989) stated that in 8 dogs diagnosed with this defect, 7 were brachycephalic dogs [33]. Fossum (2007) and Hassan (2018) also indicate that pectus excavatum frequently occured in brachycephalic dogs [1, 3]. An important factor included by that the authors of this paper was a retrospective study that encompassed dogs of the selected brachycephalic breeds, with radiographic examinations of the thorax, regardless of the clinical symptoms. However, pectus excavatum may give no significant clinical symptoms and may be detected accidentally, particularly if the sternal deformity is minor [1, 2, 4]. This is also related to the phenomenon described in human medicine [34], where the defect is fairly easy to detect in a clinical examination, but often remains ignored. This study did not reveal a greater risk of PE occurrence in dogs of group A as compared to to those of group B. However, while the presence of PE was observed in dogs of all the studied breeds, the greatest risk of PE occurrence was found in two breeds: Maltese and English Bulldog (60% and 58.3%, respectively). Such a high percentage of dogs with diagnosed PE should indicate a necessity to re-evaluate the hereditary nature of this defect, as suggested by other authors [3, 5, 7]. To date it has not been possible to identify the genes responsible for the development of PE in dogs [7, 16, 30, 33]. While the Bulldog is frequently mentioned as a breed at risk of PE, the presence of this defect in Maltese and King Charles Spaniel is reported for the first time, as far as the authors are aware.

Another thoracic wall defect, pectus carinatum, is only sporadically discussed in veterinary literature, probably due to the common belief that this defect produces no clinical symptoms in animals [2, 35]. To the authors' knowledge, respiratory distress was reported merely one dog suffering from PC, and then only when it was being exercised [9]. This study demonstrated that while PC occurred four times less frequently than PE in the brachycephalic dog group, it is not as rare as previously believed. It was demonstrated that there was a possibility of increased PC risk in screw-tailed brachycephalic dogs, the breeds at particular risk were: Pug and French Bulldog. This information is important, as a genetic predisposition for this defect has been demonstrated in humans. It has also been found that almost a quarter of humans with PC have a family history of thorax wall deformity, thoracic vertebrae deformity, congenital heart disease or marfan syndrome [11]. Further studies involving a larger number of dogs may demonstrate how great a danger PC is for the health of Pug and French Bulldog breeds.

The results showed that deformities of the thoracic spine were observed in 27.4% of the study dogs, particularly frequently in English and French Bulldog breeds. This result was similar to the authors' expectations. The literature frequently addresses the issue of the predisposition of brachycephalic dog breeds, especially screw-tailed ones, to spine malformations [13, 14, 36]. The study also confirmed a stronger, 25-times higher risk of the occurrence of kyphosis in the thoracic spine sections in dogs of screw-tailed brachycephalic breeds. Other spine malformations (scoliosis, kyphoscoliosis) were noted rarely, and only in group A.

In this study, tracheal hypoplasia was observed in 25% of the study animals. Tracheal hypoplasia is one of the primary anatomic components of Brachycephalic Airway Syndrome [37]. It is believed that brachycephalic dog breeds are loaded with this defect, particularly English Bulldog, Boston Terrier and Boxer [38], although it is reported in other dog breeds as well [39, 40]. It was demonstrated in this study that the risk of tracheal hypoplasia in screw-tailed brachycephalic breeds is five times higher than in normal-tailed brachycephalic breeds. However, the results of this study differ slightly from those reported elsewhere. Particularly notable is the low percentage of English Bulldogs (12.9%) in the animal group loaded with tracheal hypoplasia, at four times lower than in the results published by Coyne in 1992 [38]. This may be the effect of the breeder's efforts to eliminate this defect from the Bulldog population. The higher percentage of tracheal hypoplasia found in Pug and French Bulldog breeds, compared to English Bulldog, is all alarming. This observation suggests that action is required to eliminate this tracheal defect in these breeds as well.

The results indicate that heart displacement to the left or right occurred in 7.3% of the dogs in the study group. As sternal deformity may affect heart position, and in turn lead to incorrect diagnosis of cardiomegaly, or dorsal displacement of the heart, the authors decided not to include a heart size or dorsal displacement assessment [4]. Abnormal positioning of the heart was first described in detail by Suter in 1984 [39]. However, the authors did not report any statistical studies concerning the prevalence of lateral heart displacement in dogs, especially of the brachycephalic breeds. While this defect was observed twice as frequently in screw-tailed brachycephalic breeds, it was not demonstrated that dogs of group A carried a greater risk of its occurrence than group B.

The tests failed to confirm the second hypothesis, which stipulated that there was a statistically significant relation between the presence of PE or PC and thoracic spine deformity, tracheal hypoplasia or lateral heart displacement. In the available veterinary literature, information about the co-occurrence of PE and vertebral deformities in dogs of brachycephalic breeds is often quoted [1, 3–5, 7, 15]. The coexistence of spine deformities (especially scoliosis) with PE or PC is often noted in humans as well [10–12, 41]. In this study, 25.4% of the dogs with PE and 50% of the dogs with PC were also diagnosed with vertebral deformities, primarily kyphosis. The results appeared clinically significant, but not statistically significant. Some authors believe that acquired pectus excavatum may be a result of an upper airway disease and inspiratory dyspnoea [1, 8]. Suter [2] presents an opinion to the contrary, which this study also confirms. While PE was detected in 35.5% of dogs diagnosed with tracheal hypoplasia, no statistically significant relation between the two was demonstrated. Unlike the results of other authors [1, 5, 7, 15, 33], lateral cardiac displacement was the least frequently observed defect to accompany PE (12.7%). According to some studies, lateral heart displacement can occur in over 50% of the dogs affected by PE [3, 33]. It is possible that the cause of this discrepancy is that this study covered all animals with PE diagnosed by radiological tests, regardless of whether the disease was accompanied by clinical symptoms. Another discrepancy in the results of different studies concerns the issue of whether heart displacement to the left or right is more common in dogs suffering from PE. In humans, it is believed that local sternal convexity towards the inside of the thorax leads to leftward displacement and axial rotation of the heart [34]. The observations of Sutter [2] and Fossum [33], as well as the results of this study, appear to support this hypothesis. However, according to Hassan [3] and Kurosawa [8], the heart is always displaced to the right in dogs with PE. However, Rahal recorded two dogs, where in one the heart was displaced to the right and in the other to the left. It is possible that the statistical analysis may explain such a large discrepancy in the observations by different authors. These observations indicated that there was no statistically significant relation between the occurrence of PE and lateral heart displacement in brachycephalic dog breeds. Also no such statistically significant relation was found in dogs carrying PC.

It is interesting that these results demonstrated the risk of leftward heart displacement in dogs of brachycephalic breeds, as well as the presence of tracheal hypoplasia, is more likely related to the presence of kyphosis, and not PE. The risk of tracheal hypoplasia was approximately two and a half times greater in dogs suffering from thoracic spine kyphosis. A similar relation was found between thoracic spine kyphosis and heart displacement to the left. The authors found no papers concerning any correlation between vertebral deformity and lateral heart displacement or tracheal hypoplasia. Suter partially explains this issue [39] noting that if the spinal deformation makes one hemithorax significantly smaller while enlarging (stretching) the other, the heart is always displaced toward the larger one. Nevertheless, according to Suter, it is a matter of thoracic wall deformation, similar to PE. In this study, however, no relation between lateral heart displacement and the presence of PE was found. This indicates that the issue needs further study.

The retrospective nature of the present study applies some limitations on the results. Above all, the study encompassed a relatively low number of animals. Further multicentre studies may enable a more accurate assessment of the prevalence of pectus excavatum and pectus carinatum in more brachycephalic dog breeds. Another limitation is that the study was based only on radiograms of the thorax. Tomographic analysis of the thorax would likely enable a more thorough analysis of the changes caused by PE and PC, as well as an assessment of any thorax asymmetry resulting from PE or vertebral deformity, and their impact on heart position.

## Conclusion

The goal of this study was to evaluate the prevalence of PE and PC, thoracic spine deformities, tracheal hypoplasia, lateral heart displacement in screw-tailed and normal-tailed brachycephalic dog breeds. The prevalence of PE in dogs of brachycephalic breeds was 44%, and PC was 11.3%. In conclusion dogs of screw-tailed brachycephalic breeds carry a greater risk of deformity of thoracic vertebrae, tracheal hypoplasia, compared to "normal-tailed" breeds. No such relation was demonstrated for PE. However, there is a possibility of a higher risk of PC occurrence in screw-tailed breeds. It was demonstrated that the greater risk of PE occurrence was carried by the Maltese and the English Bulldog, and PC by the Pug and the French Bulldog breeds. The relations between presence of PE or PC and the other studied anomalies were not proved. It was demonstrated that in dogs of brachycephalic breeds there was a greater risk of co-incidence between kyphosis in thoracic spine and lateral heart displacement, as well as kyphosis in thoracic spine and tracheal hypoplasia.

## Supporting information

S1 Appendix Sample analysis of the relations between the PC thoracic defect risk factor and leftward cardiac malposition in the R statistical software. (CRAN Project).
(DOCX)

S1 Table Raw data from chest radiography. ok—no abnormalities, number sternebrae—place of the sternum deviation, CA kyph—Kyphosis, Cobb angle, CA scol—Scoliosis, Cobb angle, TH/TI—ratio of tracheal diameter to thoracic inlet distance.
(XLSX)
