# Supplementary material for: Prevalence of pectus excavatum (PE), pectus carinatum (PC), tracheal hypoplasia, thoracic spine deformities and lateral heart displacement in thoracic radiographs of screw-tailed brachycephalic dogs
Source: PLoS One. 2019 Oct 10;14(10):e0223642. doi: 10.1371/journal.pone.0223642 (PMC6786652; doi:10.1371/journal.pone.0223642)
Supplement: S1 Appendix — (CRAN Project). (DOCX) [file pone.0223642.s001.docx]

S~~2~~1. **Sample analysis of the relations between the PC thoracic defect risk factor and leftward cardiac malposition in the R statistical software** (CRAN Project).

Tab X Number of dogs in individual groups:

Code in the R CRAN Project statistical software (the # character denotes a comment line ignored by the program):

a<-1; a_0_<-a_1_<-b_0_<-b_1_<-a

#initial data for the Bayesian MCM simulation - variables are assigned the value of 1 #information about selecting the uniform distribution a priori.

y_1_<-0; y_0_<-14; n_1_<-2; n_0_<-67

#assignment of values to variables, representing the numbers of specimens assigned to #individual y_1_, y_0_, n_1,_ n_0_

p_0_ <- rbeta(10000, y_0_+a_0_, n_0_+b_0_)

#deriving posterior distribution p_0_ for: 10000 samples in MCMC simulation with the parameter of # beta distribution α=y_0_+a_0_, β=n_0_+b_0_

p_1_ <- rbeta(10000, y_1_+a_1_, n_1_+b_1_)
#deriving posterior distribution p_1_ for: 10000 samples in MCMC simulation with the parameter of # beta distribution α=y_1_+a_1_, β=n_1_+b_1_

OR <- p_1_*(1-p_0_)/(p_0_*(1-p_1_))

#formula OR

mean(OR); quantile(OR, c(0.025, 0.975))

#showing values of mean and CI for p=95%.
